# Integrated Analysis of Microbiome and Serum Metabolomics to Understand How *Rauvolfia vomitoria* Alkaloids Inhibit Colorectal Cancer Growth

**DOI:** 10.3390/pharmaceutics18101272

**Published:** 2026-10-07

**Authors:** Yanli Zhang, Zhifan Wang, Weiliang Cui, Jianfeng Wang, Shuqi Wang

**Affiliations:** 1 Department of Pharmacy, Qilu Hospital of Shandong University, Jinan 250012, China; yanlizhangqlyy@163.com; 2 School of Pharmaceutical Science, Shandong University, 44# West Wenhua Road, Jinan 250012, China; wangzhf_sunset@163.com; 3 Shandong Institute for Food and Drug Control, Jinan 250101, China; xinyao2002@126.com; 4 Department of Pain Management, Qilu Hospital of Shandong University, Jinan 250012, China

**Keywords:** *Rauvolfia vomitoria* alkaloids, 5-FU, colorectal cancer, gut microbiota, lipid metabolism

## Abstract

**Background/Objectives**: *Rauvolfia vomitoria* alkaloids (RVA) exhibited excellent activity against colorectal cancer (CRC) growth in the heterotopic model. However, their antitumor effects in the orthotopic models, as well as the underlying mechanisms related to gut microbiota and serum metabolomics, remain poorly understood. **Methods**: We performed 16S rRNA sequencing and serum metabolomics analysis in orthotopic CRC-bearing mice following different treatments. Univariate and multivariate logistic regression analyses were used to screen key gut microbes and metabolites associated with RVA treatments. KEGG enrichment analysis was conducted to identify the metabolic pathways modulated by RVAs in vivo. **Results**: RVA, either alone or in combination with 5-Fluorouracil (5-FU), exhibited a potent inhibitory effect on CRC growth, comparable to intraperitoneally administered 5-FU. In terms of body weight and organ indices, RVA showed lower toxicity compared to 5-FU. Both treatments induced measurable shifts in the gut microbial profiles of CRC mice. Increased abundance of *Akkermansia* and *Lactobacillus* and decreased abundance of *Enterococcus* were found to be associated with RVA monotherapy. The enrichment of *Acinetobacter* and *Mucispirillum* in CRC mice was suppressed by the combination treatment. A serum metabolomics study revealed that linoleic acid metabolism and β-oxidation of long-chain fatty acids were modulated by both RVA-containing regimens. **Conclusions**: These findings demonstrate that oral administration of RVA, either alone or combined with current clinical therapeutic methods, may represent a promising strategy for CRC treatment. Targeted modulation of gut microbiota and fatty acid metabolism may provide promising approaches to hinder CRC progression.

## 1. Introduction

Globally, colorectal cancer (CRC) is the third most commonly diagnosed cancer and the second leading cause of cancer-related deaths [1]. The intestinal microenvironment, a complex system including immunocytes, fibroblasts, blood vessels, and cytokines, plays an important role in CRC initiation and progression. Since approximately 70% of the body’s microorganisms reside in the colon, gut microbiota homeostasis, which is readily affected by stress, medications, diet, and lifestyle, is crucial to maintaining a healthy intestinal microenvironment [2]. Gut microbiota dysbiosis may trigger chronic inflammation and ultimately result in the occurrence of cancer [3].

Different gut microbes exert diverse effects during CRC carcinogenesis. Enrichment of enterotoxigenic *Bacteroides fragilis*, *pks+ Escherichia coli*, *Streptococcus gallolyticus* and *Morganella morganii* and depletion of *Akkermansia muciniphila*, *Streptococcus thermophilus*, *Streptococcus salivarius*, *Lactobacillus gallinarum*, *Clostridium butyricum* and *Carnobacterium maltaromaticum* were observed in CRC patients [4,5,6]. The underlying molecular mechanisms by which these bacteria and their metabolites act are complex and have been widely studied over the past decade. For example, the genotoxin cytolethal distending toxin produced by pathogenic *pks^+^ Escherichia coli* and *Campylobacter jejuni* could mediate DNA damage by inducing double-strand breaks [7,8], while the *Bacteroides fragilis* toxin causes genomic instability in colonic cells by inducing the production of reactive oxygen species (ROS) [9]. Other mechanisms such as modulation of inflammation and immune responses have also been demonstrated [10,11]. Several beneficial bacteria confer anticancer effects through various mechanisms, such as fermentation supernatants of *Lactobacillus delbrueckii* through a caspase 3-dependent pathway, ferrichrome via JNK-mediated apoptosis and reuterin through altering redox balance [12,13,14]. Additionally, *Lactobacillus kefiranofaciens* ZW18 can enhance the antitumor effect of anti-programmed cell death-1 (PD-1) immunotherapy [15]. Collectively, these findings corroborate the critical roles of the intestinal microbiota in CRC development and treatment, highlighting the need to develop corresponding targeted therapeutic strategies.

Despite the availability of a variety of treatment strategies for CRC, such as chemotherapy, surgery, immunotherapy and radiotherapy, the incidence of adverse effects remains high, affecting the patient’s quality of life and increasing the death rates [16,17]. Recently, traditional Chinese medicine (TCM) has been widely utilized in cancer treatment because of its merits of low cost, excellent antitumor activity and convenient administration [18]. Oral administration of TCM inevitably brings these agents into contact with intestinal bacteria. Therefore, TCM offers a promising option for CRC treatment via modulation of the gut microbiota and related metabolic pathways. In previous studies by our group, we screened a series of TCM extracts for their inhibitory effect on CRC growth. The results showed that *Rauvolfia vomitoria* alkaloids (RVA) extract, either alone or combined with 5-Fluorouracil (5-FU), had potent anti-CRC effects, as demonstrated both in vitro and in mice bearing subcutaneous xenografts. *Rauvolfia vomitoria* is a rich source of monoterpene indole alkaloids, such as reserpine, ajmalicine, ajmaline, serpentine and yohimbine, which displayed fascinating novel chemical structures and excellent biological activities against different cancers, such as CRC, ovarian cancer, lung cancer and pancreatic cancer [19,20,21,22,23,24], making them an attractive source for the discovery of new drugs. Previously, we performed a serum metabolomics study on the mechanism of the anti-CRC effect of RVA in combination with 5-FU in mice bearing subcutaneous CRC xenografts and found that the lipid metabolism and cell energy metabolism pathways of the mice were modulated [25]. However, considering the limitations of the heterotopic model, the role of gut microbiota in CRC progression associated with RVA use has not been investigated yet. Moreover, the close link between gut microbiota and host metabolism highlights the need to comprehensively understand how RVA inhibits CRC growth through integrated multi-omics approaches.

In the present study, we evaluated the therapeutic effects of RVA, administered alone and in combination with 5-FU, in an orthotopic CRC mouse model and investigated the underlying mechanisms. We profiled the alterations in the gut microbiota and metabolites following different treatments, thereby demonstrating the treatment-associated gut microbiota dysbiosis and serum metabolic reprogramming. These findings may facilitate TCM applications in CRC treatment, elucidate their underlying mechanism, and identify novel promising therapeutic targets.

## 2. Materials and Methods

### 2.1. Plant Materials

The roots of *Rauvolfia vomitoria* were collected in Nanning, Guangxi Province, in February 2018, and authenticated by Professor Xiaoning Wang, from the College of Pharmacy, Shandong University, China. The specimen is deposited in the Drug Analysis and Testing Center of the College of Pharmacy, Shandong University, with the accession number RVWN-2018002. RVA was prepared and characterized in our lab as described previously [26]. Generally speaking, the powdered dried roots were extracted with 95% ethanol (250 g/L) three times, followed by acidification with 5% H_2_SO_4_ to pH 1.0 and extraction three times with petroleum ether and ethyl acetate, respectively. Finally, the resultant aqueous layer was neutralized with 10% NaOH solution and extracted with CH_2_Cl_2_ to yield RVA. The contents of five main alkaloids, namely reserpine, yohimbine, ajmaline, ajmalicine and sarpagine, in RVA were determined using HPLC-MS/MS to be 4.7, 7.04, 26.03, 26.51 and 25.33 mg/g, respectively [27].

### 2.2. Reagents

Mouse CT26 CRC cells were purchased from Cellverse Co., Ltd. (Shanghai, China). RPMI 1640 medium, 0.25% trypsin, and fetal bovine serum were obtained from Gibco Corporation (New York, NY, USA). 5-FU was purchased from APExBIO (Houston, TX, USA). Formalin solution, hematoxylin and eosin were obtained from Servicebio Technology Co., Ltd. (Wuhan, China). Other chemicals, including acetonitrile, anhydrous ethanol, dimethyl benzene, and n-butanol, were of analytical grade.

### 2.3. Animal Experiments

Healthy male SPF-grade BALB/c mice, weighing 18.22 g and aged 6 weeks (animal license SCXK (Jing) 2021-0006), were purchased from Vital River Laboratories Animal Technology Co., Ltd. (Beijing, China). The mice were housed under the following conditions: temperature of 22 ± 2 °C and humidity of 50 ± 10%. All the animal experiments were approved by the Ethical Committee of the School of Pharmaceutical Sciences, Shandong University, and conformed to the basic principles of animal experimentation (approval code: 22050).

To establish the orthotopic CRC mouse model, healthy male BALB/c mice were anesthetized. CT26 wide-type cells were trypsinized, washed twice with sterile normal saline, and suspended in sterile normal saline to a final concentration of 4 × 10^7^ cells mL^−1^. The cell suspension was then transferred into a sterile tube and gently triturated with a sterile pipette, followed by subcutaneous injection of 100 μL of the cell suspension into the right axilla of each mouse using a 1 mL syringe. All operations were completed within 30 min to preserve tumor cell viability. Mice were monitored daily for general health, and distinct palpable tumors developed in the right axilla on day 10 post-inoculation. Once tumors reached approximately 1 cm in diameter, the mice were euthanized, and the tumor was aseptically harvested via axillary skin incision with ophthalmic scissors and cut into uniform 1 mg (~1 mm^3^) fragments using sterile surgical scissors under an stereomicroscope (Olympus Corporation, Tokyo, Japan). These fragments were subsequently affixed to the cecal serosa. Laparotomy was then performed to exteriorize the cecum, and 1 mm^3^ CRC tissue was affixed to the abraded intestinal mucosa using surgical tissue adhesive. Finally, the abdomen was closed and disinfected with 75% ethanol. Body weight and tumor growth of the mice were recorded every day. Three days after the tumor inoculation, palpable tumor nodules were detected in the abdominal cavity of all mice, and no signs of infection were observed.

**Figure 1:**
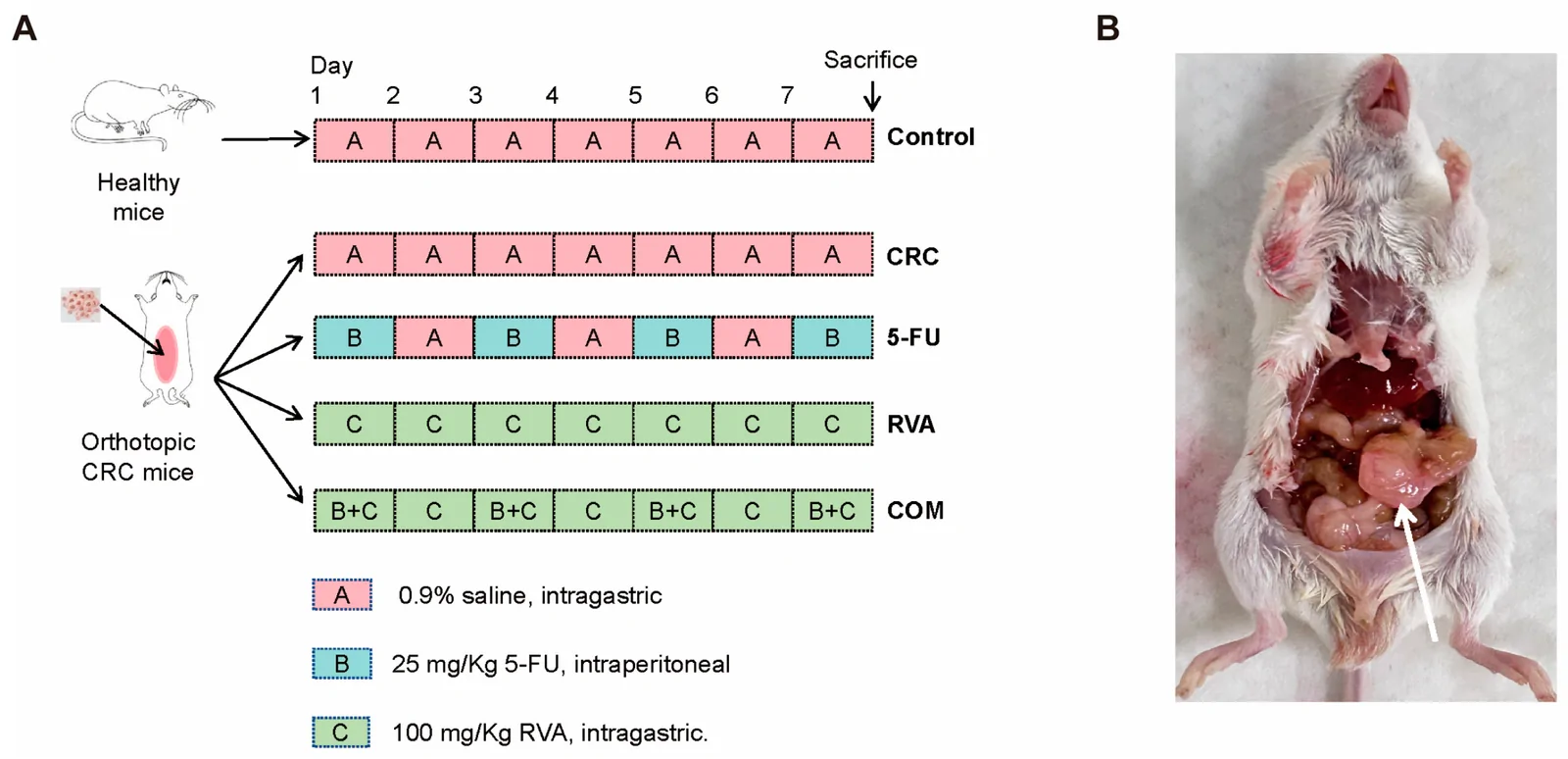
A flow chart of the details of the animal experiment. (**A**) The mice were divided into five groups: Control, CRC, 5-FU, RVA and COM groups; (**B**) at the end of the experiment, the mice were sacrificed and dissected. The white arrow indicates the orthotopic CRC tumor.

Following tumor implantation and confirmation of engraftment, the mice were randomly assigned to five groups (*n* = 6 per group), including the Control, CRC model, 5-FU, RVA and COM groups (Figure 1). Randomization was conducted by a researcher independent of subsequent experimental procedures using a computer-generated random number sequence to ensure unbiased group assignment. In the study, healthy mice were used as controls to evaluate the toxicity of different treatments. CRC-bearing mice receiving no treatment or 5-FU monotherapy were used to assess the antitumor effects of RVA and the combination treatment. All control groups received the same treatment schedule as the experimental groups. Moreover, all mice were housed and handled under identical conditions. Throughout the experiment, the investigators who performed health assessments and tumor measurements of the mice were blinded to group assignments and treatments.

After the final drug administration on the 7th day, the mice were fasted for 12 h. To collect the stool samples, the mice were placed in a clean cage, and their abdomens were gently massaged to stimulate defecation. Fresh fecal samples were collected and stored at −80 °C for further analysis.

Blood samples were collected via eyeball enucleation immediately after anesthesia. Freshly collected blood was allowed to stand on ice for 2-3 h, then was centrifuged at 4 °C and 3000 rpm for 15 min. The supernatant was collected as serum samples. For the untargeted metabolomics analysis, a total of 600 μL pre-cooled acetonitrile was added to 200 μL mouse serum samples for protein precipitation. The mixture was then vortexed for 1 min, left to stand at room temperature for 10 min, and then centrifuged at 4 °C and 14,000 rpm for 10 min. Then, 500 μL of the supernatant was dried under nitrogen gas at 37 °C. Prior to sample analysis, the residue was reconstituted with 100 μL acetonitrile–water (4:1, *v*/*v*), followed by centrifugation at 4 °C and 14,000 rpm for 10 min. An aliquot of 80 μL supernatant was transferred into an autosampler vial for analysis. Ten-microliter aliquots of each supernatant were pooled to prepare quality control (QC) samples for system calibration. Finally, the mice were euthanized by cervical dislocation after isoflurane inhalation and dissected. The weights of heart, liver, spleen, kidney, and tumor tissues were recorded. The organ index (%) was calculated as the ratio of organ weight to body weight multiplied by 100 according to the previous literature [28].

### 2.4. 16S rRNA Sequencing and Analysis

Total fecal DNA was extracted using the Fecal DNA Mini-Prep Kit according to the manufacturer’s instructions (Guangzhou Magen Biotechnology Co., Ltd., Guangzhou, China). Using diluted genomic DNA as the template, with 341F (5′-CCTACGGGNGGCWGCAG-3′) and 806R (5′-GGACTACHVGGGTATCTAA-3′) as primers, the V3-V4 region of the bacterial 16S rRNA gene was amplified [29]. The PCR products were purified with AMPure XP Beads and quantified using the ABI Step One Plus Real-Time PCR System (Thermo Fisher, Massachusetts, MA, USA). DNA library sequencing was performed on the Illumina NovaSeq 6000/PacBio Revivo platform by Gene Denovo Biotechnology Co., Ltd. (Guangzhou, China). Bioinformatic analysis was performed using Omicsmart, a dynamic real-time interactive online platform for data analysis (http://www.omicsmart.com, accessed on 1 September 2023).

Alpha diversity analysis was performed to characterize the richness and evenness of species within the groups. Commonly used alpha diversity indexes include Sobs, Chao1, Shannon, and Simpson. A large index indicates great diversity within the group. β-diversity analysis, such as principal coordinate analysis (PCoA) and non-metric multidimensional scaling (NMDS), is used to describe the differences across groups. The farther the distance between different groups, the greater the difference between the groups. The abundance of gut microbiota in each group was statistically analyzed at multiple taxonomic levels, and significantly different taxa were identified [30].

### 2.5. Untargeted Metabolomics Study

The detailed procedures for sample preparation, HPLC-MS/MS experiments, and generation of the peak-ion intensity matrix were described in our previous publication [25]. Generally, for HPLC, the experiments were set up as follows. Chromatographic column: Thermo Hypersil GOLD C18 (2.1 mm × 100 mm, 1.9 μm) (Thermo Fisher, Massachusetts, MA, USA); mobile phase A, 0.1% formic acid in water; mobile phase B, 0.1% formic acid in acetonitrile; flow rate, 0.3 mL/min; injection volume, 5 μL; and column temperature, 40 °C. For MS, sample analysis was performed in both positive and negative ion modes by electrospray ionization (ESI) under the following conditions: spray voltage, 3.5 kV for positive ions and 2.8 kV for negative ions; capillary temperature, 325 °C; sheath gas, 35 arb; auxiliary gas, 10 arb. The metabolomics data were collected using full MS/dd MS2 (Top10) mode. Data acquisition and processing were performed using Thermo Xcalibur 4.0 software.

For metabolite identification, the MS/MS original data files were first imported into Compound Discoverer 3.0 (Thermo Fisher, Massachusetts, MA, USA) and aligned by time, peak picking, noise removal, peak alignment, deconvolution, and normalization to generate a data matrix containing retention time, m/z value, and peak intensity value. Combined with the secondary mass spectrometry fragment ion information collected by MS/MS, online compound databases, including mzCloud (http://www.mzcloud.org, accessed on 9 November 2024), ChemSpider (http://www.chemspider.com, accessed on 9 November 2024), and HMDB (http://www.hmdb.ca, accessed on 9 November 2024), were then used for metabolite annotation.

Multivariate statistical analysis was performed using MetaboAnalyst 6.0 (https://www.metaboanalyst.ca/, accessed on 15 December 2025). Prior to statistical analysis, the raw metabolite intensity data underwent multi-step preprocessing. First, features with a sample-to-blank intensity ratio < 10 were discarded as noise. From the remaining features, 40% of variables with the lowest interquartile ranges (IQRs) were excluded to retain metabolically informative signals. Next, missing values were imputed using a limit-of-detection (LOD)-based left-censored approach, substituting each missing entry with one-fifth of the minimum positive intensity observed for that specific feature across all samples. Subsequently, data were normalized by dividing each sample’s intensities by its median value, followed by log_2_ transformation. Finally, prior to statistical modeling, the data were Pareto-scaled.

Unsupervised principal component analysis (PCA) was used to visualize the metabolic differences across the groups. To identify the distinct metabolite markers that distinguished the CRC from the RVA or COM treatment groups, orthogonal partial least squares discriminant analyses (OPLS-DA) were constructed. The quality of each OPLS model was evaluated with goodness of fit (*R*^2^) and its predictive ability (*Q*^2^) [31]. Models with *R*^2^ and *Q*^2^ greater than 0.5 were considered acceptable. Variable importance in projection (VIP) values were calculated to quantify the contribution of each variable to the model construction. Metabolites with VIP > 1 were screened as differential metabolites and imported to the pathway analysis module of MetaboAnalyst 6.0 for metabolic pathway enrichment analysis.

### 2.6. Statistical Analysis

In this study, GraphPad Prism 10.0 was used for statistical analysis. All data were presented as mean ± standard deviation (SD). One-way analysis of variance (ANOVA) was performed, followed by Tukey’s honestly significant difference multiple comparisons. Statistical differences were visualized using a compact letter display (CLD). Means sharing any common letter are not significantly different; means without shared letters differ significantly (*p* < 0.05). The correlation between gut microbiota and metabolites was evaluated using the Spearman rank correlation analysis for two-group comparison. Specifically, microbial genera were selected for correlation analysis according to the following criteria: they were detected in all samples of different groups; they showed significant differences between groups (*p* < 0.05). Metabolites were selected based on the following criteria: they were detected in all samples of different groups; VIP score > 1 from the OPLS-DA model, for which both *R*^2^ and *Q*^2^ are larger than 0.5. Correlation heatmaps were generated with the correlation coefficient threshold set as 0.8 and the significance cutoff at *p* < 0.05. The color gradient in the heatmap represents the value of Spearman’s correlation coefficient (r): red indicates positive correlations, while green denotes negative correlations. Asterisks in the heatmap mark the statistical significance of each pairwise association: * *p* < 0.05, ** *p* < 0.01, *** *p* < 0.001.

## 3. Results

### 3.1. RVA Suppresses Tumor Progression in Orthotopic CRC Mouse Models

The growth status of mice was observed daily. In the present study, the mental state of mice was assessed by monitoring their daily behavior indicators, including food and water intake, hair glossiness, and locomotor activity. Mice in the Control group exhibited good mental status, with normal feeding behavior and locomotor activity. By contrast, CRC-bearing mice displayed loss of weight, messy hair and decreased locomotor activity. All three treatments improved the mental status of the mice. Subsequently, all mice were weighed, sacrificed, and dissected. Tumor tissues, heart, liver, spleen, and kidney of each mouse were collected and weighed. Organ indices were calculated and statistically compared across all groups.

**Figure 2:**
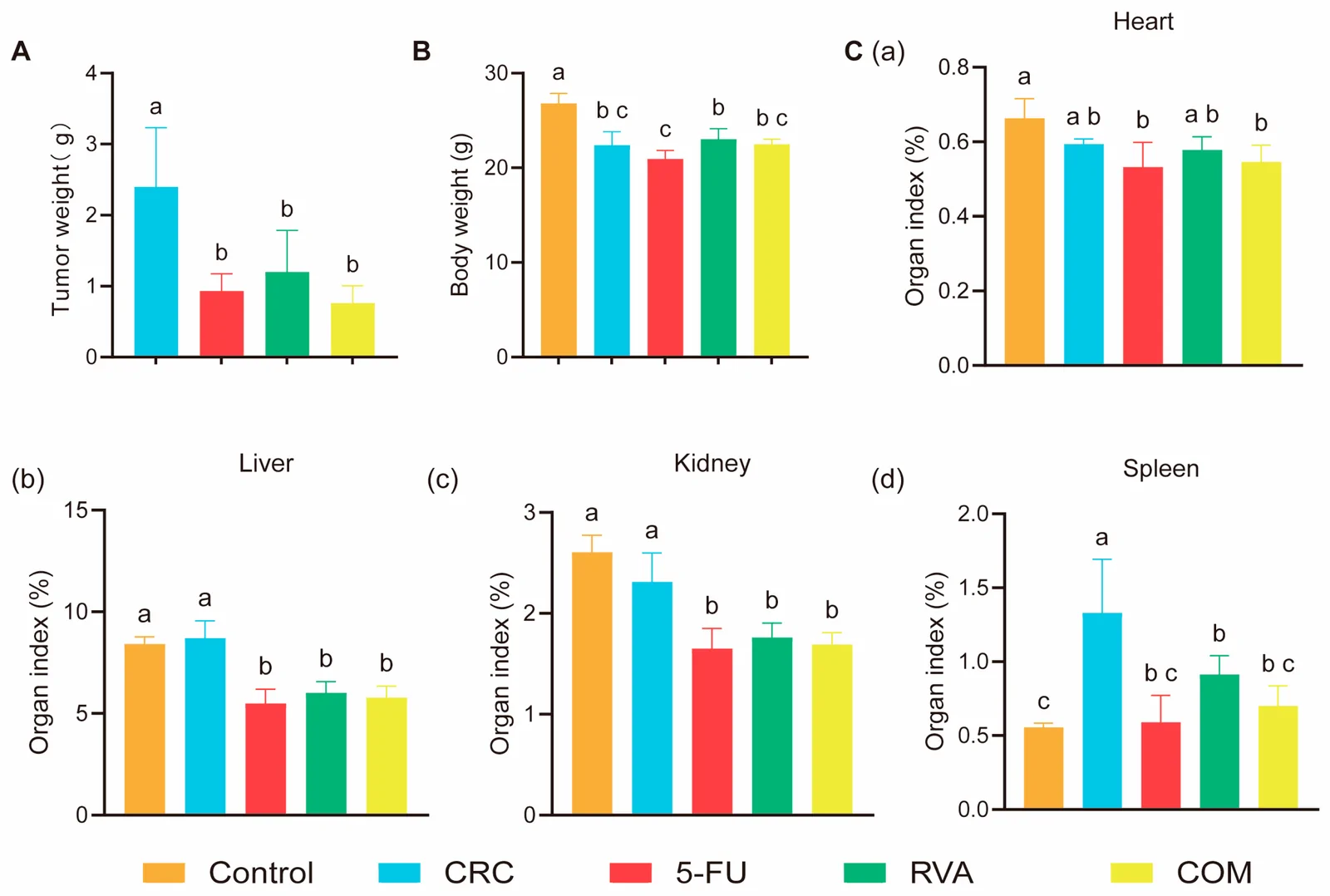
The inhibitory effect of RVA in combination with 5-FU on tumor progression in orthotopic CRC mice. All mice were sacrificed and dissected. Tumor tissues, as well as heart, liver, kidney and spleen, were harvested and weighed. Tumor weight (**A**), body weight (**B**) and organ indices (**C**) were calculated and statistically analyzed. The statistical analysis results were visualized using CLD. Means sharing any common letter are not significantly different; means without shared letters differ significantly (*p* < 0.05).

As shown in Figure 2A, all three treatments significantly suppressed tumor growth, and no significant differences were observed among the 5-FU, RVA, and COM groups. These results demonstrate that RVA possesses potent anti-CRC activity. Compared with the healthy Control mice, CRC-bearing mice showed significant body weight loss (Figure 2B). Moreover, 5-FU treatment induced additional significant body weight loss relative to the CRC model group, suggesting the existence of chemotherapeutic toxicity. In contrast, weight loss was not aggravated in mice receiving RVA monotherapy or the COM treatment.

Organ indices were then compared to evaluate drug safety (Figure 2C). No obvious differences in liver, heart and kidney coefficients were found between Control and the CRC groups. For the heart, the organ index of the CRC group was markedly higher than those of the 5-FU and COM groups, while being comparable to that in the RVA group. Notably, treatment with 5-FU, RVA or COM significantly reduced the liver and kidney organ indices when compared with Control and CRC mice, indicating drug toxicity. Furthermore, compared to the Control group, the organ index of the spleen increased significantly in the CRC group, suggesting that CRC development may cause splenic dysfunction. Administration of 5-FU, RVA or COM effectively alleviated CRC-induced splenomegaly, resulting in a significantly lower spleen index compared with the untreated CRC group. Compared to 5-FU, RVA treatment caused less toxicity in terms of both the organ indices and body weight, although the differences were not significant enough. Collectively, these results demonstrate that intragastric administration of RVA, either alone or in combination with 5-FU, effectively inhibited CRC development in the orthotopic mouse model. Its antitumor effect is comparable to that of intraperitoneal injection of 5-FU but induces milder in vivo adverse effects.

### 3.2. Overview of the Gut Microbiota in Different Groups

**Figure 3:**
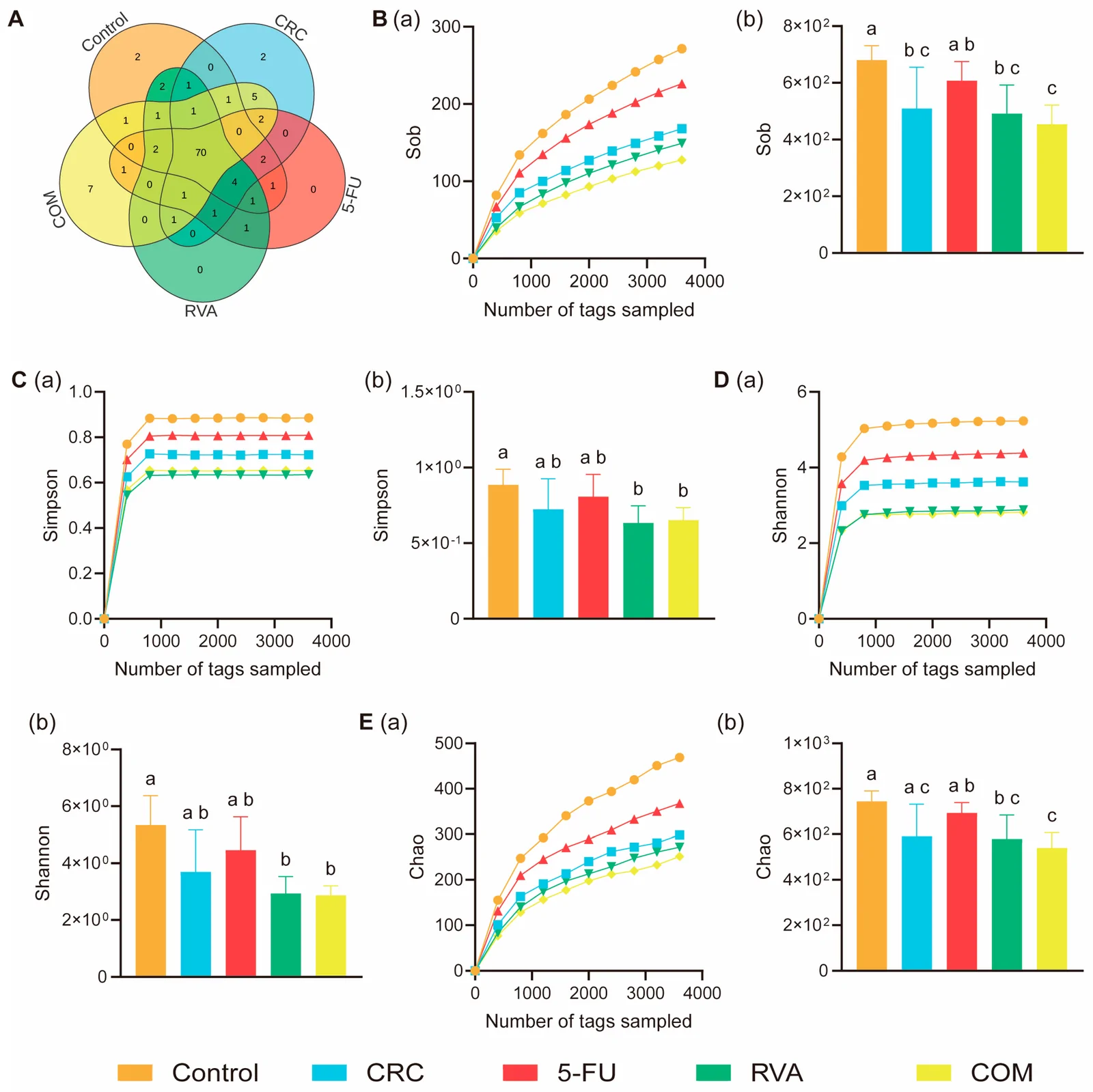
Overview of the gut microbiota across different groups. (**A**) A Venn diagram; (**B**–**E**) the dilution curves (**a**) and α-diversity analysis (**b**) for the evaluation of microbiota richness (Sobs and Chao1) and diversity (Shannon and Simpson) in different groups. ANOVA was performed, followed by Tukey’s HSD multiple comparisons. Results were visualized using CLD. Means sharing any common letter are not significantly different; means without shared letters differ significantly (*p* < 0.05).

Mouse fecal samples were subjected to 16S rRNA sequencing to analyze the intestinal microbiota composition of CRC mice following different treatments. A Venn diagram was generated to characterize the microbes shared and unique to different groups. As shown in the results (Figure 3A), at the genus level, a total of 73 genera were common to all five groups. The numbers of genera unique to the Control, CRC and COM groups were 2, 2, and 7, respectively, indicating high similarity in the gut microbiota composition across the groups.

The results of the α-diversity analysis are shown in Figure 3B–E. The rarefaction curves based on the Chao1, Sobs, Shannon and Simpson indices supported the adequacy of the sequencing depth and amount for all the samples. Subsequent α-diversity statistical analysis results demonstrated that the Sobs index was significantly lower in the CRC group compared with the Control group (*p* < 0.05). In contrast, no significant differences in Simpson, Shannon and Chao indices were observed between the Control and CRC mice. Treatment with 5-FU partially restored the reduced α-diversity induced by CRC. Nevertheless, RVA and COM groups displayed markedly lower Sobs, Simpson, Shannon and Chao indices relative to the Control group, with no significant differences from the CRC group. No significant differences in α-diversity were detected between RVA and COM groups.

### 3.3. Alterations in the Gut Microbiota Composition Associated with RVA Treatment

**Figure 4:**
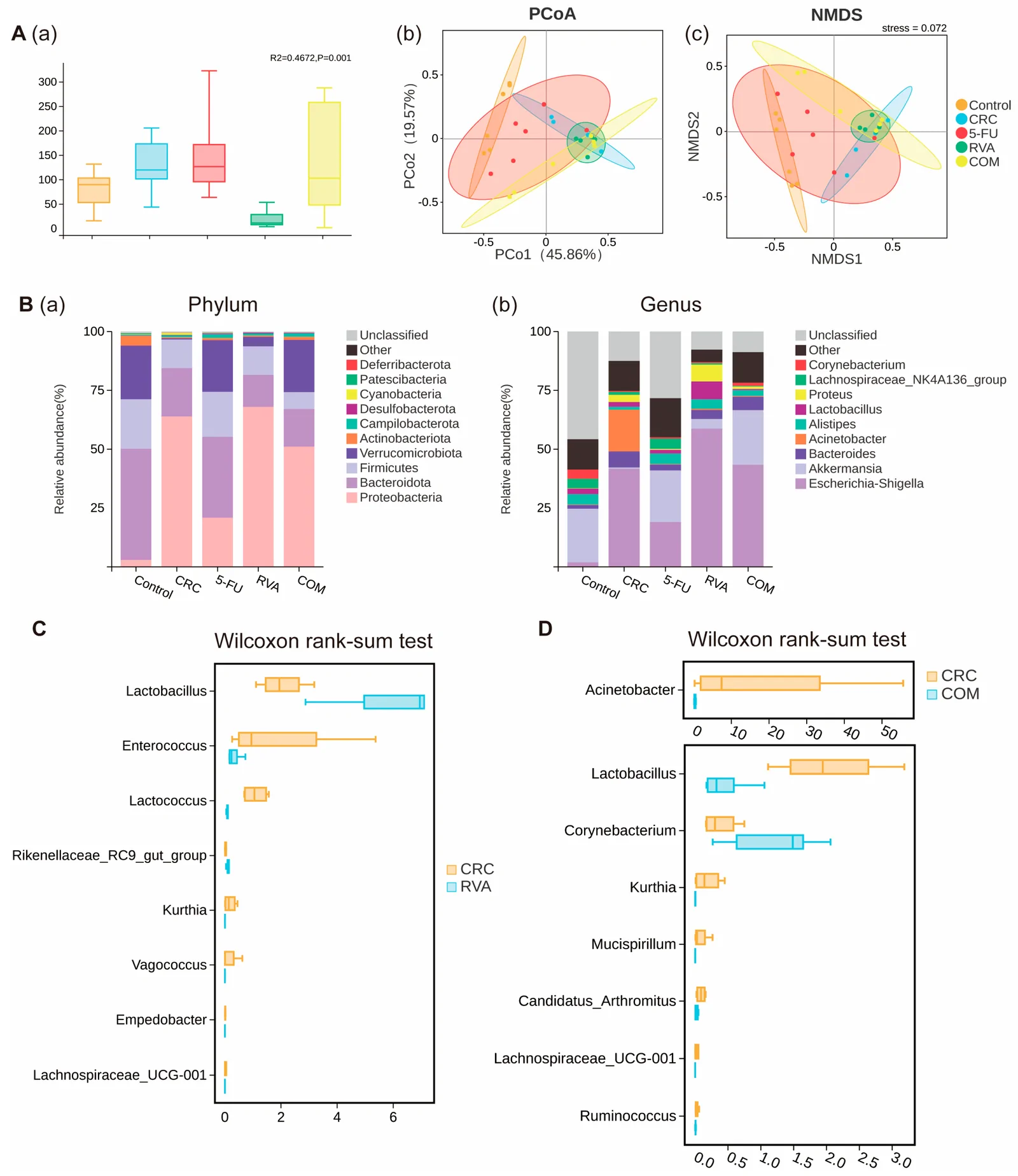
Alterations in the composition of fecal microbiota detected by 16S rRNA sequencing associated with RVA and COM treatments. (**A**) β-Diversity analysis of the gut microbiota: ordination plots based on the Adonis test (**a**), NMDS (**b**) and PCoA (**c**) using Bray–Curtis characterized the taxonomic variations in microbial communities across the five groups; (**B**) relative abundance of gut bacteria at the phylum (**a**) and genus (**b**) levels; Wilcoxon rank-sum test to examine the significant differences in genus between CRC versus RVA (**C**) and CRC versus COM (**D**) (*p* < 0.05).

β-diversity analysis was performed to investigate the patterns of gut microbiota across all groups. The Adonis test verified that the overall bacterial community structure differed significantly across all experimental groups (Figure 4A). As visualized in both NMDS (stress = 0.072) and PCoA ordination plots based on Bray–Curtis distance, clear separation of the gut microbial community was observed between the Control and CRC groups (Figure 4A). The top two axes of PCoA collectively explained 65.43% of the total variance in microbiota composition. None of the treatment groups fully restored the gut microbiota profile to resemble that of the healthy Control mice.

To screen the microorganisms that were significantly changed upon RVA treatment, species composition analysis was performed at two different taxonomic levels, as shown in Figure 4B. At the phylum level, *Proteobacteria*, *Bacteroidota*, *Firmicutes* and *Verrucomicrobiota* were the top four enriched phyla in the fecal microbiota. In the CRC group, the relative abundance of *Proteobacteria* increased significantly, while those of *Verrucomicrobiota* and *Bacteroidota* decreased significantly compared to the Control group. Intervention with 5-FU, RVA or COM partially reversed this dysbiosis. At the genus level, *Escherichia-Shigella*, a typical pro-inflammatory pathobiont, was enriched in the CRC group compared with the Control group. 5-FU treatment effectively reduced its abundance, while its growth was not inhibited by RVA and COM treatments. Another opportunistic pathogen, *Acinetobacter,* was also upregulated in the CRC mice, and all three treatments suppressed its overgrowth. Moreover, RVA monotherapy significantly enriched beneficial *Lactobacillus* and *Proteus*. *Akkermansia* was abundant in the Control group but sharply depleted after CRC modeling. Both 5-FU and COM treatments effectively restored its population. However, RVA alone showed limited capacity to recover *Akkermansia*. We would like to clarify that only the top 10 most abundant bacterial taxa are visualized in this figure, as these predominant taxa are presumed to contribute more substantially to gut environmental homeostasis. Apart from these dominant taxa, at the phylum level, the other bacteria include *Chloroflexi, Synergistota, Euryarchaeota*, and *Armatimonadota*. At the genus level, there are additional 59 identifiable bacterial taxa with relative abundance above 0.1%. Given the large number of these low-abundance taxa, it would be impractical to display all of them clearly within the figures. We have added a Supplementary Table S1 to list these taxa assigned to the “other” category, presenting their detailed relative abundance information (Table S1).

Wilcoxon rank-sum tests were performed to investigate the effect of RVA and COM treatments on the gut microbiota composition (Figure 4C,D). Taxa with *p* < 0.05 were regarded as significantly altered biomarkers. Relative abundance of *Lactobacillus* was significantly higher in the RVA group compared with CRC mice. Conversely, multiple genera enriched in the CRC cohort, including *Enterococcus*, *Lactococcus*, *Kurthia*, *Vagococcus* and *Lachnospiraceae_UCG-001*, were markedly depleted following RVA treatment. The opportunistic pathogen *Acinetobacter* displayed significantly lower abundance in the COM group than that in the CRC group. Meanwhile, the abundance of *Lactobacillus* was markedly reduced in COM mice compared with CRC mice. Multiple CRC-enriched taxa, including *Kurthia*, *Mucispirillum* and *Lachnospiraceae_UCG-001,* were suppressed upon combination treatment, whereas the level of *Corynebacterium* was elevated in the COM group. Collectively, all three therapeutic regimens induced observable shifts in the gut microbial profiles of CRC mice. Nevertheless, RVA monotherapy and COM combination treatment shaped the gut microbiota in a distinct manner compared with 5-FU alone, indicating that these agents may exert anti-colorectal cancer effects through divergent microbiota-associated mechanisms.

### 3.4. Changes in the Serum Metabolomics Features Associated with RVA Administration

**Figure 5:**
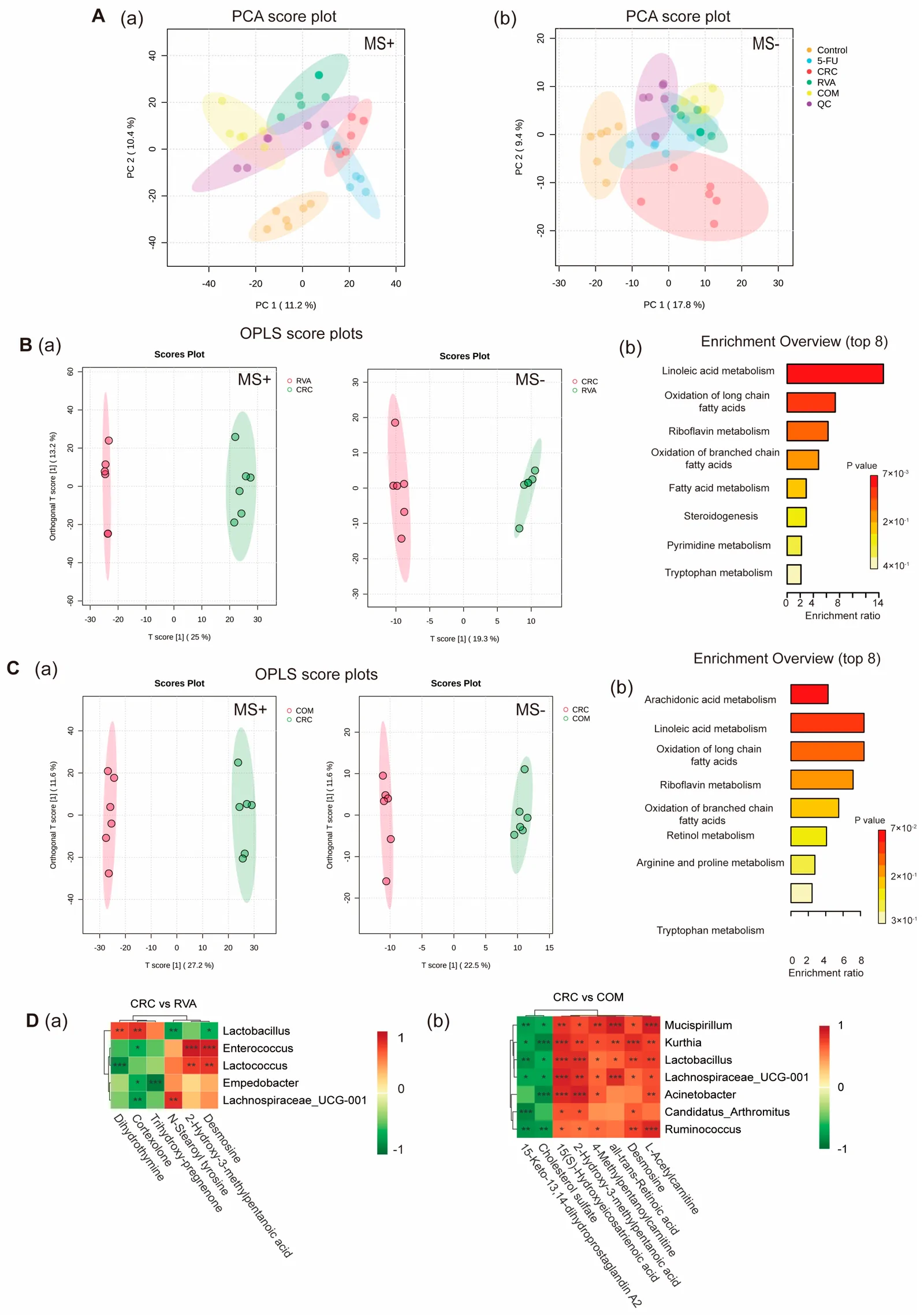
Changes in the serum metabolomics features associated with RVA and COM treatments. (**A**) PCA score plots showing clustering of the five groups for both (**a**) positive and (**b**) negative modes; (**B**) (**a**) OPLS-DA score plots and (**b**) pathway enrichment analysis results of significantly changed metabolites between CRC and RVA groups; (**C**) (**a**) OPLS-DA score plots and (**b**) pathway enrichment analysis results of significantly changed metabolites between CRC and COM groups; (**D**) (**a**) correlations between differential gut taxa and differential metabolites related to RVA administration; (**b**) correlations between differential gut taxa and differential metabolites related to combination administration. The color gradient in the heatmap represents the value of Spearman’s correlation coefficient (r): red indicates positive correlations, while green denotes negative correlations. Asterisks in the heatmap mark the statistical significance of each pairwise association: * *p* < 0.05, ** *p* < 0.01, *** *p* < 0.001.

Given the crosstalk between the gut microbiota and host metabolic pathways, the serum metabolomics analysis was conducted through the untargeted HPLC-MS/MS method. PCA based on untargeted metabolomics data was performed under positive ion mode (MS+) and negative ion mode (MS−) to evaluate global metabolic variations across the five experimental groups (Figure 5A). Quality control (QC) samples were closely aggregated, demonstrating good stability and reproducibility of the LC-MS detection system. Clear separation was observed between the Control group and the CRC group in both ion modes. Samples from the 5-FU, RVA and COM groups exhibited distinct distributions compared with the CRC group. The metabolic profiles of RVA and COM samples showed different features from those of the 5-FU group.

OPLS-DA models satisfying the criteria of *R*^2^ > 0.5 and *Q*^2^ > 0.5 were constructed for pairwise comparisons (CRC vs RVA, CRC vs COM) in both modes, aiming to identify differential metabolites associated with the treatments (Figure 5B,C). Using the VIP score > 1, *p* < 0.05, as the threshold, a total of 65 metabolites were found to be significantly changed in the RVA group compared with the CRC group. To identify the metabolic pathways affected by RVA in the CRC mice, pathway enrichment analysis based on KEGG human metabolic pathways was performed, and the results showed that the most influenced pathways included linoleic acid metabolism, β-oxidation of long-chain fatty acids, and riboflavin metabolism. For the CRC vs. COM comparison, a total of 74 metabolites were found to be significantly changed. The top three influenced pathways included arachidonic acid metabolism, linoleic acid metabolism, and β-oxidation of long-chain fatty acids.

A Spearman correlation analysis was performed using the Metware Cloud (https://cloud.metware.cn, accessed on 15 September 2026). Heatmaps were generated to visualize the associations between the regulated microbial genera and metabolites (Figure 5D). For CRC vs RVA, five bacterial genera and seven differential metabolites formed two distinct correlation clusters. *Lactobacillus* showed significant positive correlations with steroid-related metabolites including dihydrothymine and cortexolone (*p* < 0.01), whereas it was negatively correlated with N-stearoyl tyrosine (*p* < 0.01) and desmosine (*p* < 0.05). *Enterococcus* and *Lactococcus* were significantly positively correlated with desmosine and 2-hydroxy-3-methylpentanoic acid, yet negatively associated with steroid-derived metabolites. *Empedobacter* exhibited strong negative correlations with trihydroxy-pregnenone (*p* < 0.001). Lachnospiraceae_UCG-001 was positively correlated with N-stearoyl tyrosine (*p* < 0.01) and negatively correlated with cortexolone (*p* < 0.01). For CRC vs COM, two distinct metabolite clusters were observed. Metabolites, including 15-keto-13,14-dihydroprostaglandin A_2_ and cholesterol sulfate, exhibited widespread significant negative correlations with most tested genera (*Mucispirillum*, *Kurthia*, *Lactobacillus*, *Lachnospiraceae_UCG-001*, *Acinetobacter*, *Candidatus_Arthromitus*, *Ruminococcus*). In contrast, metabolites such as 15(S)-hydroxyeicosatrienoic acid, all-trans-retinoic acid, desmosine and L-acetylcarnitine showed extensive positive correlations with these bacterial taxa.

**Figure 6:**
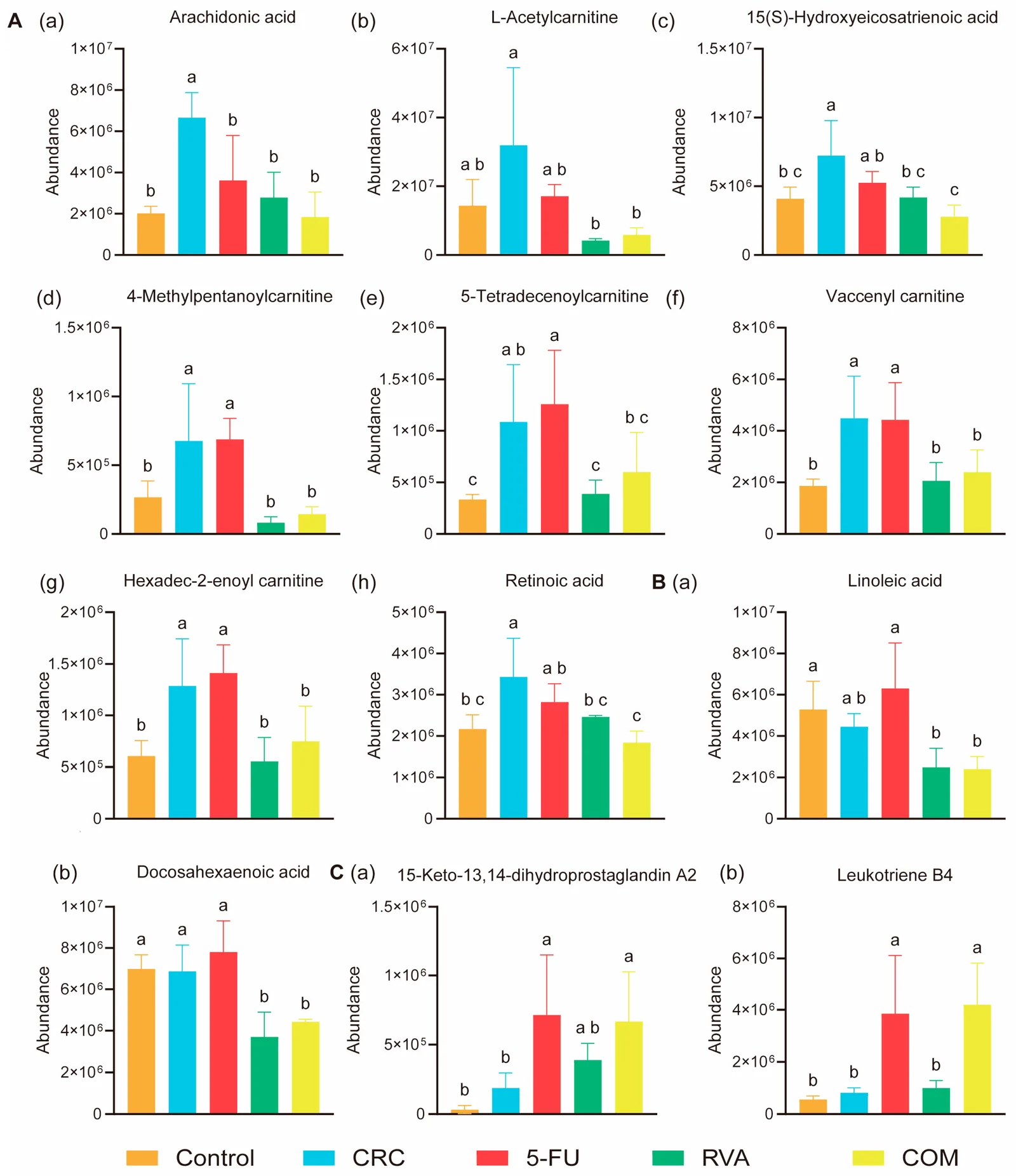
Serum levels of differentially abundant metabolites across the five groups. ANOVA was performed, followed by Tukey’s HSD multiple comparisons. (**A**) Serum levels of arachidonic acid, multiple acylcarnitines and retinoic acid; (**B**) serum levels of linoleic acid and docosahexaenoic acid; (**C**) serum levels of 15-keto-13,14-dihydroprostaglandin A2 and leukotriene B4. Results were visualized using CLD. Means sharing any common letter are not significantly different; means without shared letters differ significantly (*p* < 0.05).

Quantitative alterations of the differential metabolites closely associated with the aforementioned metabolic pathways were further characterized. Compared with the Control group, the CRC group exhibited significantly elevated levels of arachidonic acid, multiple acylcarnitines and retinoic acid (Figure 6A). RVA monotherapy and COM combination therapy significantly reduced these upregulated metabolites, while such effects were not observed in the 5-FU group. As shown in Figure 6B, no significant differences in linoleic acid and docosahexaenoic acid were observed between the Control group and CRC group. However, significantly lower levels of linoleic acid and docosahexaenoic acid were detected in the COM and RVA groups when compared with the CRC group. Figure 6C illustrates the alterations of two key inflammatory eicosanoids, 15-keto-13,14-dihydroprostaglandin A2 and leukotriene B4. Compared with the Control and CRC groups, treatment with 5-FU and COM markedly increased the abundances of both metabolites, whereas no significant difference was detected between the RVA and CRC groups. Collectively, RVA and COM exerted regulatory effects on lipid metabolic profiles of CRC mice in a manner distinct from 5-FU monotherapy.

## 4. Discussion

Plant-derived alkaloids have always been an important source of antitumor drugs, such as vincristine and camptothecin, which have shown excellent performance in the treatment of gastrointestinal tumors [32,33]. Therefore, searching for compounds with excellent antitumor activity from traditional medicines rich in alkaloids constitutes a core strategy for novel drug discovery. Previously, our group has optimized the protocol for preparation of RVA and established an LC-MS-based analytical method for the quantification of major alkaloids. Moreover, the inhibitory activity of RVA against CRC has been previously demonstrated using CRC cells and in mice with CRC subcutaneous xenografts, and its possible mechanism of action, interference with lipid metabolism, has been revealed from the metabolic perspective. However, in mice with CRC subcutaneous xenografts, the real intestinal state of the mice, which plays an important role in drug absorption and efficacy, especially when most TCM were administered via oral administration, is usually neglected.

In this study, we successfully constructed an orthotopic CRC mouse model, which provided a more appropriate model to mimic CRC growth in the intestinal environment [34], to evaluate the anti-CRC activity of RVA. Accumulating evidence has confirmed the great contribution of gut microbiota to CRC progression and its response to therapy. Meanwhile, the relationship between TCM and the intestinal flora has attracted lots of attention because of their strong and various interactions [35,36]. Therefore, this study systematically explored the mechanism of RVA against CRC from the perspective of gut microbiota and metabolic pathways in order to identify new therapeutic strategies and targets.

In the orthotopic CRC mouse model, both RVA monotherapy and combination therapy with 5-FU demonstrated potent inhibitory effects on CRC progression comparable to those of 5-FU monotherapy, alleviated the CRC-induced splenic dysfunction, and induced moderate toxicity to the liver and kidneys. Notably, in terms of body weight and cardiac toxicity, RVA exhibited superior performance compared with 5-FU monotherapy. Nevertheless, this conclusion must be interpreted with caution. It is important to acknowledge that body weight, behavioral observations, and organ coefficients cannot detect subclinical organ injuries (e.g., early hepatocellular damage or renal tubular dysfunction) that can only be reliably captured by serum biochemical markers. To definitively address this question in future investigations, we will incorporate dedicated toxicology cohorts with standardized clinical chemistry panels, including serial measurements of alanine aminotransferase (ALT) for hepatic function, creatinine and blood urea nitrogen (BUN) for renal function, and cardiac troponin for myocardial injury, at multiple pre-defined time points. Such a systematic approach will be essential to rigorously compare the safety windows and organ-specific toxicity profiles between the two regimens in subsequent dose-escalation studies.

Among the five major alkaloids quantified in the RVA, reserpine has been proven to be able to suppress the growth and angiogenesis of human HCT116 colon tumors in immunodeficient mice by inhibiting intercellular biomolecule transfer [37]. Integrated bioinformatic analysis of distant metastasis-related genes and candidate therapeutic agents in colon adenocarcinoma further predicted that ajmaline acts as a promising anti-metastatic candidate by targeting prognosis-relevant metastatic genes [38]. However, the antitumor activity of the other three alkaloids has not been reported yet. Our previous research established a ^1^H NMR-based biochemometric flow and verified that alkaloids of lower content, such as lochnerine, 1-methyl-β-carboline and sarpagine, also exhibited good inhibitory effects against HCT-8 and LoVo CRC cells [39]. Thus, the potent antitumor activity of RVA extract is most likely attributed to additive and partially synergistic bioactivity of its diverse alkaloid constituents.

Gut microbiota homeostasis plays a crucial role in CRC initiation and progression, rendering it a promising therapeutic target for CRC treatment. In the present study, all three treatments modulated the gut microbiota in orthotopic CRC-bearing mice, but their regulatory modes differed greatly. The gut microbial composition of 5-FU-treated mice shifted toward that of healthy mice. However, RVA and COM induced characteristic microbial profiles that differed from both the Control and CRC groups, indicating divergent microbiota-regulating mechanisms between 5-FU and RVA-associated treatments.

All three treatment groups displayed a higher abundance of *Akkermansia* than the CRC group. As the major species in the genus *Akkermansia*, *A. muciniphila* resides in the gut of various animals and plays a crucial role in maintaining intestinal homeostasis [40]. Its abundance can be affected by multiple factors, such as different intestinal diseases, diet and lifestyles; therefore, it has a close relationship with CRC development and treatment. Consistent with the finding of decreased abundance of *A. muciniphila* in the fecal microbiota of CRC patients [41], we also observed an identical depletion trend in our orthotopic model. An acetyltransferase isolated from *A. muciniphila* can blunt colorectal tumorigenesis by reprogramming the tumor microenvironment. It can induce HSP70 secretion and promote CTL-related immune response [42]. Furthermore, a study using an azoxymethane/dextran sodium sulfate (AOM/DSS) mouse model demonstrated that *A. muciniphila* attenuates the progression of CRC by inhibiting tryptophan metabolism via the AhR/β-catenin signaling pathway [43]. Therefore, *A. muciniphila* has long been regarded as a promising next-generation probiotic in targeting intestinal health. However, dietary supplementation with *A. muciniphila* requires precise regulation because research shows that an increase in *A. muciniphila* and its metabolic product succinate increased the susceptibility of the intestine to *Citrobacter rodentium* infection [44].

Of note, unlike the other two treatment groups, RVA treatment failed to fully restore the relative abundance of *Akkermansia*, suggesting the existence of other modulatory mechanisms. Previous research showed that reduced *Lactobacillus animalis* abundance in mice with gastrointestinal dysmotility induced the accumulation of linoleic acid, thereby increasing colitis susceptibility [45]. Therefore, we infer that the enrichment of *Lactobacillus* in the RVA group may contribute to its anti-CRC effects by preventing the accumulation of pro-carcinogenic metabolites like linoleic acid. *Lactobacillus* strains also exert multiple protective roles against CRC. For instance, by recruiting dendritic cells, *Lactobacillus rhamnosus*, *Lactobacillus johnsonii* and *Lactobacillus intestinalis* attenuated CRC tumorigenesis through their effects on the immune microenvironment [46]. Moreover, indole-3-lactic acid produced by *Lactobacillus plantarum* exerted an antitumor effect when combined with anti-PD-1 treatment [47].

RVA treatment caused a significant reduction in the abundance of *Enterococcus*, which may confer protective effects against CRC. Previous studies have demonstrated that *Enterococcus faecalis* induced DNA damage in epithelial cells by promoting superoxide production and facilitated CRC progression via inducing inflammation and accumulation of additional mutations and epigenetic changes [48]. Its metabolites biliverdin and tyramine can also increase CRC risk and accelerate angiogenesis in CRC by increasing DNA damage and cell proliferation and inhibiting cell cycle arrest [49,50]. In the COM group, the relative abundance of *Mucispirillum* was significantly lower than in the CRC group. *Mucispirillum* schaedleri is also known to be a pro-inflammatory bacterial species and is closely linked to the development of Crohn’s-disease-like colitis in rodents [51]. Therefore, the suppression of *Acinetobacter* and *Mucispirillum* abundance by the combination therapy might partially explain its capacity to alleviate CRC-associated inflammatory disorders. Overall, the modulation of gut microbiota associated with RVA use in the CRC mice is rather complicated and hence may exert anticancer effects as a whole via modulation of metabolic functions in vivo.

Untargeted serum metabolomics analysis was then performed to investigate the changes in metabolite levels influenced by RVA-associated treatments. Two metabolic pathways, including linoleic acid metabolism and β-oxidation of long-chain fatty acids, were disturbed in both RVA and COM groups, compared with the CRC group. Further investigation of the differentially abundant metabolites revealed that the upregulation of arachidonic acid and multiple acylcarnitines induced by CRC progression was suppressed by the RVA and COM treatments, highlighting probable lipid-metabolic regulatory roles underlying their antitumor activity. Arachidonic acid and its metabolites regulate inflammatory responses critical to CAC onset and progression [52]. Meanwhile, the accumulation of diverse acylcarnitines reflects hyperactive mitochondrial fatty acid β-oxidation (FAO) in CRC cells. Pharmacological or natural compound-mediated suppression of acylcarnitine biogenesis suppressed tumor growth by counteracting lipid oxidative damage [53,54].

A significant downregulation of linoleic acid and docosahexaenoic acid upon RVA-associated treatments was also observed. Linoleic acid, as a representative of omega-6 polyunsaturated fatty acids, widely exists in food [55]. A meta-analysis of prospective studies involving 787,490 participants showed that dietary intake of linoleic acid was associated with an increased CRC risk, despite a lack of studies on the underlying mechanisms [56]. Long-term treatment of cancer cells with linoleic acid has been found to induce a dormancy-like phenotype of cancer cells, which tend to be treatment-resistant [57]. The inhibitory effects of RVA monotherapy and combination therapy on the aforementioned metabolites indicate them as potential therapeutic targets, and future mechanistic assays are still required to clarify the underlying molecular mechanisms. Furthermore, 5-FU and COM groups displayed significantly higher levels of two key inflammatory eicosanoids, 15-keto-13,14-dihydroprostaglandin A2 and leukotriene B4, whereas no such change was detected in the RVA monotherapy, indicating that the two metabolites might be related to the chemotherapeutic toxicity of 5-FU.

Microbe–metabolite correlation analyses provided potential mechanistic insights into the gut-associated effects of the RVA and its combination with 5-FU-based chemotherapy (COM) in CRC. Distinct microbe–metabolite correlation architectures were observed in CRC vs RVA and CRC vs COM comparisons, implying that monotherapy and combined chemotherapy may exert anti-CRC properties by remodeling divergent gut microbiota–metabolite axes. Under RVA monotherapy, *Lactobacillus* exhibited robust associations with steroid-related metabolites, whereas *Enterococcus*, *Lactococcus*, *Empedobacter*, and *Lachnospiraceae_UCG-001* were predominantly correlated with organic acid and amino acid-derived metabolites. Intestinal steroid metabolism exerts critical regulatory effects on colonic epithelial proliferation and mucosal inflammatory homeostasis [58]. Accordingly, it is plausible that the anti-tumor activity of RVA may be partially attributable to remodeled crosstalk between *Lactobacillus* and intestinal steroid metabolic cascades. In comparison to RVA monotherapy, combination treatment (COM) reconfigured the microbe–metabolite interaction toward a substantially different profile. The majority of identified genera displayed negative correlations with 15-keto-13,14-dihydroprostaglandin A_2_ and cholesterol sulfate, alongside broad positive coupling with eicosanoid mediators, all-trans-retinoic acid, as well as the acylcarnitine family, L-acetylcarnitine and 4-methylpentanoylcarnitine. Given that 5-FU-based chemotherapy frequently induces intestinal mucosal damage and excessive eicosanoid-mediated inflammatory responses, these correlative observations support the hypothesis that COM may reprogram gut microbial composition to modulate pro-inflammatory metabolic signaling and lipid metabolism, which may collectively contribute to the therapeutic benefits of combinatorial intervention [59]. Notably, *Lactobacillus* and *Lachnospiraceae_UCG-001* were detected in both correlation datasets yet engaged in divergent metabolic networks under monotherapy versus combination regimens. This finding indicates that the metabolic outputs of these commensal taxa are context-dependent and can be profoundly modulated by the co-administration of 5-FU [60]. It should be emphasized that all above-mentioned relationships are correlative rather than causal. Correlation alone does not establish a biological or mechanistic relationship. Further experimental validation, such as microbiota-depleted animal models and in vitro bacterial incubation assays, is required to verify the functional roles of these candidate microbe–metabolite pairs in mediating the therapeutic effects of RVA and COM.

Despite these promising results, this study still has certain limitations. First, it was conducted in a limited number of mouse samples, which, while valuable, may not be enough to provide convincing support for the RVA application in CRC treatment. Further investigations, which cover a large number of samples, as well as human populations, are needed to validate these findings. Second, deeper validation of the mechanistic pathway is lacking in the current study, for instance, growth curve experiments to validate the effects of RVA on the growth of identified microbe species and bacterial production of metabolites; microbiome-depletion experiments (e.g., antibiotic treatment) to investigate the role of identified microbe species in CRC development; and the relationship between linoleic acid metabolism and the identified microbe species. Finally, the precise molecular mechanisms through which RVA modulates the gut microbiota and exerts its effects on host metabolic pathways remain unclear and should be elucidated in the future.

## 5. Conclusions

In summary, the present study systematically investigated the anti-CRC efficacy and underlying mechanisms of RVA monotherapy and its combination with 5-FU using an orthotopic CRC mouse model. Compared with our previous study, the significant advancement of the present study is as follows. First, an orthotopic CRC mouse model was established and employed. Compared to subcutaneous xenograft models, it offers many advantages, such as higher physiological relevance and stronger clinical relevance. Especially for the discovery of oral formulations for CRC treatment, a model that can mimic the real CRC intestinal environment and the interaction between drugs and tumor tissues is rather meaningful. Second, the gut microbiota plays an important role throughout the entire process of CRC development and therefore understanding its interaction with different treatments is crucial for the discovery of potential clinical drugs. We have, for the first time, found that RVA treatment caused alterations in the microbiota composition of CRC mice and proposed an idea that gut microbiota might be an important target of RVA in CRC treatment. Third, we also investigated the effect of RVA-associated therapy on the metabolism of CRC mice and found that the treatments suppressed the accumulation of pro-carcinogenic linoleic acid and regulated key metabolic pathways, including linolenic acid metabolism, arachidonic acid metabolism and fatty acid beta-oxidation. Overall, this work confirms that RVA exerts potent anti-CRC activity through a gut microbiota–metabolism regulatory axis, offering a novel potential adjuvant strategy for CRC clinical treatment. Nevertheless, this study remains observational in nature. Further mechanistic validation and functional experiments in animal models are required to establish definitive causal relationships between RVA-mediated microbiota-metabolic reprogramming and CRC suppression.

## Data Availability

The raw LC-MS/MS data from the untargeted metabolomics analysis has been deposited in the icloud of Shandong University, and the link is “https://icloud.sdu.edu.cn/link/AA1EF03B1279F84A53AA57AD3286B24AAF, accessed on 11 September 2026”. The file name is “Integrated analysis of microbiome and serum metabolomics for understanding how Rauvolfia vomitoria alkaloids inhibit colorectal cancer growth”.

## Supplementary Materials

The following supporting information can be downloaded at: https://www.mdpi.com/article/10.3390/pharmaceutics18101272/s1, Table S1: 1. The full list of gut bacteria at both the levels of Phylum and Genus.

## Abbreviations

The following abbreviations are used in this manuscript:

RVA	alkaloid extract from *Rauvolfia vomitoria*
CRC	colorectal cancer
5-FU	5-Fluorouracil
HPLC-MS/MS	high-performance liquid chromatography-tandem mass spectrometry
TCM	traditional Chinese medicines
PCoA	principal coordinate analysis
NMDS	non-metric multidimensional scaling
ESI	electrosp ionization
PCA	principal component analysis
OPLS-DA	orthogonal partial least Squares discriminant analysis
VIP	variable importance for the project
SD	standard deviation
ANOVA	one-way analysis of variance
PD-1	programmed cell death-1
CLD	compact letter display
AOM/DSS	azoxymethane/dextran sodium sulfate
ALT	alanine aminotransferase
BUN	blood urea nitrogen
