# Supplementary material for: Integrated Analysis of Microbiome and Serum Metabolomics to Understand How Rauvolfia vomitoria Alkaloids Inhibit Colorectal Cancer Growth
Source: Pharmaceutics. 2026 Oct 7;18(10):1272. doi: 10.3390/pharmaceutics18101272 (PMC13708475; doi:10.3390/pharmaceutics18101272)
Supplement: Supplementary file 1 [file pharmaceutics-18-01272-s001.zip › pharmaceutics-4486347-supplementary.pdf]

Supplementary information

Table S1. The full list of gut bacteria at both the levels of Phylum and Genus.

**Phylum**	
Proteobacteria	Campilobacterota
Bacteroidota	Desulfobacterota
Verrucomicrobiota	Cyanobacteria
Firmicutes	Patescibacteria
Actinobacteriota	Deferribacterota
**Genus**	
Escherichia-Shigella	Butyricimonas
Akkermansia	Roseburia
Bacteroides	Enterorhabdus
Alistipes	Aerococcus
Acinetobacter	Clostridium_sensu_stricto_1
Lactobacillus	Kurthia
Lachnospiraceae_NK4A136_group	Solibacillus
Proteus	Eubacterium_nodatum_group
Corynebacterium	Intestinimonas
Citrobacter	Monoglobus
Odoribacter	Mucispirillum
Helicobacter	Faecalibaculum
Staphylococcus	Candidatus_Arthromitus
Enterococcus	Facklamia
Parabacteroides	Comamonas
Prevotellaceae_UCG-001	Flavonifractor
Erysipelatoclostridium	ASF356
Alloprevotella	Vagococcus
Muribaculum	A2
Lactococcus	Empedobacter
Rikenella	Anaeroplasma
Eubacterium_fissicatena_group	Tuzzerella
Blautia	UCG-005
Parasutterella	Candidatus_Stoquefichus
Sphingobacterium	Lachnospiraceae_UCG-001
Colidextribacter	Pseudomonas
Lachnoclostridium	Ruminococcus_torques_group
Candidatus_Saccharimonas	Jeotgalicoccus
Desulfovibrio	Anaerotruncus
Prevotellaceae_NK3B31_group	Stenotrophomonas
Incertae_Sedis	Bifidobacterium
Romboutsia	Clostridium_innocuum_group

Ruminococcus	Turicibacter
Oscillibacter	Rikenellaceae_RC9_gut_group
